# Immunometabolism in the tumor microenvironment and its related research progress

**DOI:** 10.3389/fonc.2022.1024789

**Published:** 2022-10-31

**Authors:** Ziheng Zhang, Yajun Hu, Yuefeng Chen, Zhuoneng Chen, Yexin Zhu, Mingmin Chen, Jichu Xia, Yixuan Sun, Wenfang Xu

**Affiliations:** ^1^ Medical School, Shaoxing University, Shaoxing, China; ^2^ Hubei Key Laboratory of Diabetes and Angiopathy, Hubei University of Science and Technology, Xianning, China; ^3^ Department of Clinical Laboratory, Shaoxing University affiliated Hospital, Shaoxing, China

**Keywords:** tumor microenvironment, glucose metabolism, lipid metabolism, Tregs, macrophages, NK cells

## Abstract

The tumor immune microenvironment has been a research hot spot in recent years. The cytokines and metabolites in the microenvironment can promote the occurrence and development of tumor in various ways and help tumor cells get rid of the surveillance of the immune system and complete immune escape. Many studies have shown that the existence of tumor microenvironment is an important reason for the failure of immunotherapy. The impact of the tumor microenvironment on tumor is a systematic study. The current research on this aspect may be only the tip of the iceberg, and a relative lack of integrity, may be related to the heterogeneity of tumor. This review mainly discusses the current status of glucose metabolism and lipid metabolism in the tumor microenvironment, including the phenotype of glucose metabolism and lipid metabolism in the microenvironment; the effects of these metabolic methods and their metabolites on three important immune cells Impact: regulatory T cells (Tregs), tumor-associated macrophages (TAM), natural killer cells (NK cells); and the impact of metabolism in the targeted microenvironment on immunotherapy. At the end of this article,the potential relationship between Ferroptosis and the tumor microenvironment in recent years is also briefly described.

## 1 Composition and characteristics of tumor microenvironment

**Figure 1:**
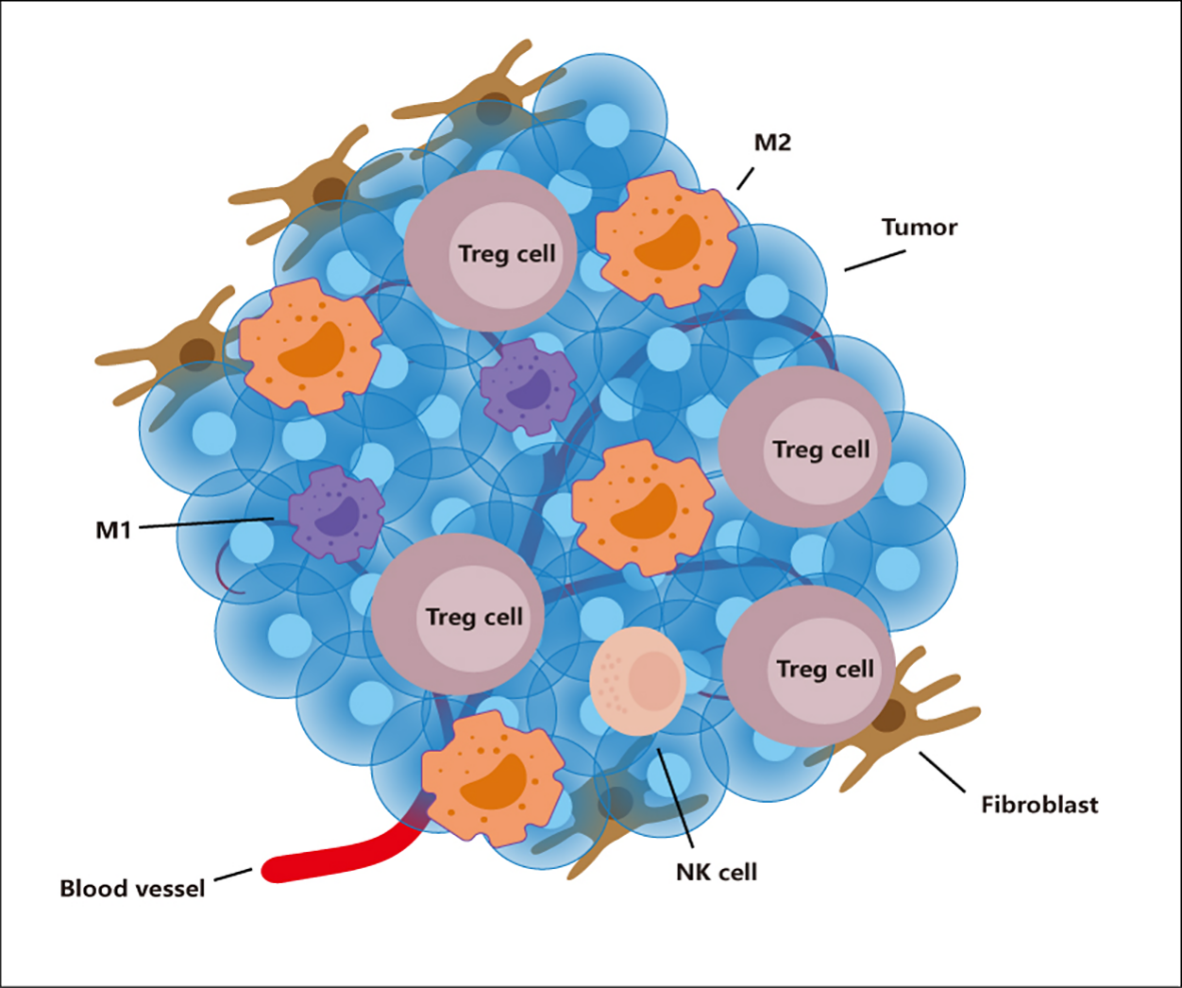
The tumor microenvironment. The tumor microenvironment contains many cells such as tumor, TAM, Tregs, NK cells, Fibroblast. Immunosuppressive cells (M2 and Tregs) show an increase in number and function in this environment. However, immune cells (M1 and NK cells) show a decrease in number and weakened function.

The tumor microenvironment is closely related to the occurrence, development, and metastasis of tumor. From the perspective of the composition of the tumor microenvironment, in addition to the tumor cells themselves, this microenvironment also includes immune cells, fibroblasts, extracellular matrix, blood vessels, et (**Figure 1**) (1). Immune cells in the tumor microenvironment can be temporarily divided into two camps. One is the immune cell camp that can effectively fight tumor, including effector T cells, NK cells, and M1 macrophages; the other is to help tumor cells get rid of immune system monitoring, the immune suppressive cell camp that promotes tumor growth, including Tregs, M2 macrophages, Myeloid-derived suppressor cells (MDSC), and resistant dendritic cells. It can be said that the immune suppressive cells that exist in the second camp are our betrayers. Because under normal circumstances, we also need these immune suppressive cells to balance the effects of the immune system, control the strength of inflammation, and prevent excessive Injury caused by inflammation. It’s just that in the tumor microenvironment, they are bought by the tumor and exert a more vital immune suppressive ability. More and more studies have shown that Tregs can kill effector T cells and antigen-presenting cells through a variety of ways: Tregs inhibits effector T cells through the PTEN-PI3K signaling pathway (2, 3); Tregs can secrete a variety of anti-inflammatory factors (IL-10, IL- 35, TGF-β) blocking the function of dendritic cells, it can also promote more Naive T cells to transform into Tregs (4–6); M2 type macrophages can combine with PD-1 on effector T cells through high expression of PD-L1, Inhibit the function of T cells (7). MDSC inhibits the function of T cells by expressing myeloid cell receptor tyrosine kinases (TYRO3, AXL, MERTK) and CD39 and CD73 (8, 9). Tolerant dendritic cells is used for T cells through PD-L1, FAS-L-mediated cell contact mechanism, and can also be used to secrete immunosuppressive cytokines like IL-10 and TGF-β which act on T cells to inhibit the function of T cells (10). Of course, this cannot be all to blame for them, because our “police”, those cells belonging to the first camp, when they enter the tumor microenvironment, are like being dissatisfied with water and soil, showing a lack of function and reduction on the quantity (11, 12). Therefore, in general, the power of the two camps in the tumor microenvironment is entirely unsuitable. This situation has also led to tumor that can get rid of the surveillance of our immune system, grow and metastasize unscrupulously. At the same time, it also brought considerable obstacles to our treatment.

So, what caused the formation of such a microenvironment? The reasons can be tentatively divided into two aspects. Many cytokines inhibit inflammation in the tumor microenvironment (13); the other is the unique metabolism of tumor cells and the impact of metabolites on immune cells (14). Clarifying the specific mechanisms that affect the tumor microenvironment in these two aspects is of great help to our supplementary immunotherapy.

## 2 Glucose metabolism in the tumor microenvironment

Growing tumor are characterized by insufficient blood attention and hypoxia (15). Hypoxia in tumor is well understood because of the constant consumption of wildly growing tumor and the lack of adequate oxygen supply. Therefore, this situation will activate the hypoxia inducible factor HIF-1 and start a series of compensatory responses. HIF-1 is composed of two subunits, HIF-1α and HIF-1β. HIF-1β plays a structural role in the protein and is stably expressed in cells. This appearance is different from HIF-1α and is not restricted by oxygen (16). While HIF-1α is an active subunit, it is usually degraded by ubiquitination under normal oxygen conditions. The typical mechanism for regulating HIF-1 is that under the condition of sufficient oxygen, the proline and asparagine on HIF-1α are hydroxylated by prolyl hydroxylase-domain protein (PHDS) and factor-inhibiting HIF (FIH). At the same time, pVHL will accumulate multiple proteins [von Hippel-Lindau tumor suppressor protein (VHL), Elongin B, Elongin C, cullin 2 (CUL2) and RING-box protein 1 (RBX1)] to form a complex, and this complex will combine with ubiquitin ligase E2 to complete the ubiquitination and degradation of HIF-1α. When under a hypoxic environment, PHDS, FIH and pVHL lose their original activity due to oxygen limitation, helping HIF-1α escape and accumulate from the fate of ubiquitination (17). In the new research, long non-coding RNA is also involved in the protection of HIF-1α under hypoxia, which can be called an atypical HIF-1 regulatory mechanism (18). Under this mechanism, HIF-1α and HIF-1β form a heterodimer and play an important role.

**Figure 2:**
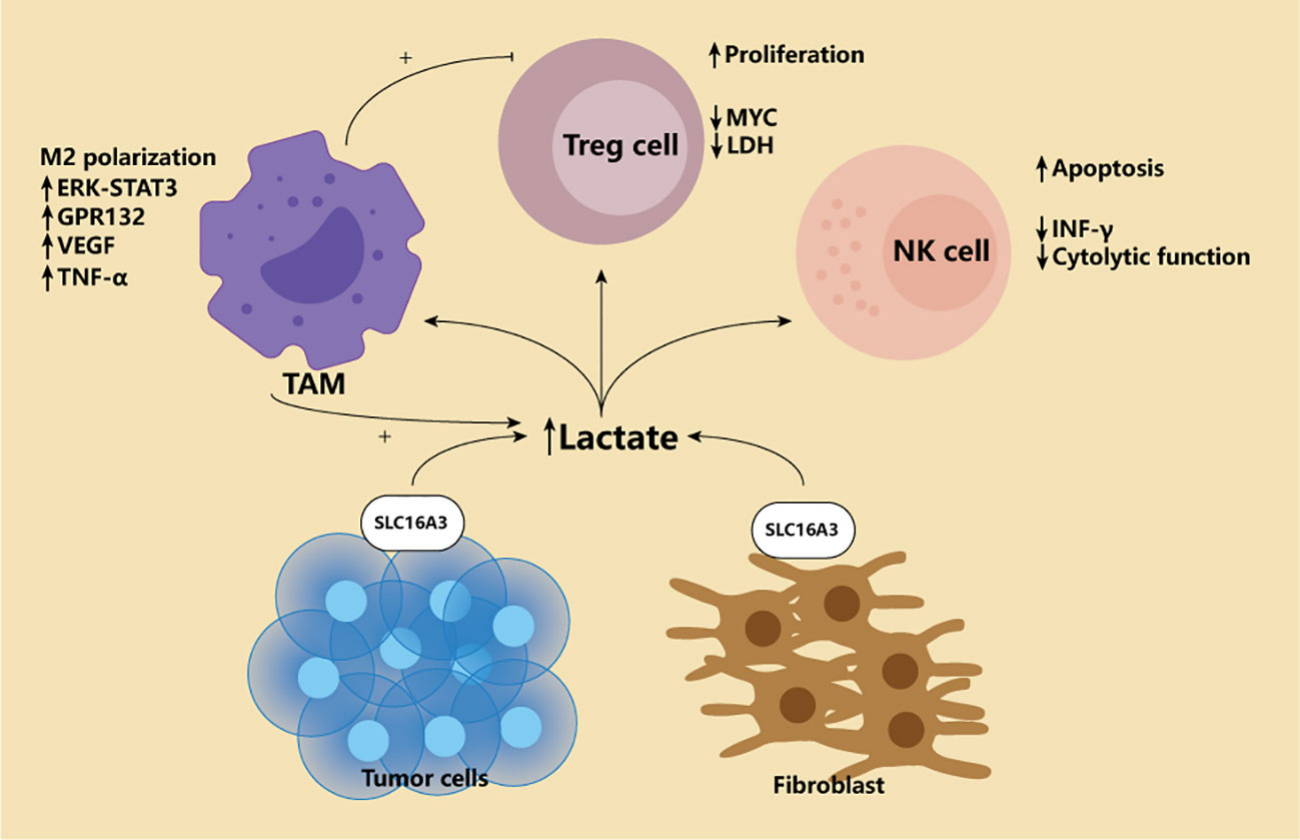
Lactate in the tumor microenvironment. Tumor microenvironment is characterized by high lactic acid enrichment. For the classic Warburg effect and the later Reverse Warburg effect, only the lactate producing cells are different. The former relies on the production of lactic acid by tumor cells themselves, while the latter relies on tumor fibroblasts. High concentration of lactic acid will affect various immune cells in the microenvironment. First, lactic acid can promote M2-type polarization of macrophages through ERK-STAT3 pathway and Gpr132. Secondly, lactic acid can also cause macrophages to overexpress VEGF, thus generating more blood vessels conducive to EMT of tumors. Finally, lactate-induced macrophages produce high levels of TNF-α, which promotes glycolysis of tumor cells, creating a vicious cycle. For Treg, high lactic acid promotes its proliferation, and Treg can actively adapt to this environment by down-regulating Myc and LDH. NK cells, on the other hand, were completely inhibited, including down-regulation of IFN-γ and cytotoxicity, and increased apoptosis.

HIF-1 is the switch of cell metabolism from mitochondrial oxidative phosphorylation to glycolysis because cells can only survive by accelerating energy production under hypoxic conditions. PI3K/AKT/mTOR pathway and HIF-1 are central regulators of glycolysis. At the same time, HIF-1 plays a vital role in regulating critical enzymes of aerobic glycolysis (19, 20). In addition, HIF-1 has also been shown to activate pyruvate dehydrogenase kinase, which converts mitochondrial oxidative phosphorylation to glycolysis by degrading the pyruvate dehydrogenase required in the tricarboxylic acid cycle (21). Many scholars, therefore, believe that the hypoxia-induced accumulation of HIF-1 in the tumor microenvironment promotes glycolysis, and metabolism is one of the reasons for the Warburg effect (22). Mammals are supposed to obtain sufficient energy through mitochondrial oxidative phosphorylation. Under aerobic conditions, the tricarboxylic acid cycle can oxidize pyruvate produced by glycolysis to carbon dioxide and produce Nicotinamide adenine dinucleotide (NADH) and FADH2, which are then made through the electron transport chain a lot of ATP. After a glucose molecule is oxidized, 36 ATP molecules can be produced. However, among tumor cells, even in an environment with sufficient oxygen, they tend to rely on glycolysis. This effect is called the Warburg effect (23). The final product of glycolysis is lactic acid produced by pyruvate through lactate dehydrogenase. During this process, each glucose molecule produces 2 ATP. In the early days, we did not know why tumor cells would use such inefficient energy production. However, with the deepening of research, researchers have discovered that the Warburg effect is not only the metabolic reprogramming of tumor cells but also helps the occurrence and development of tumor. Because tumor cells can transfer the produced lactic acid into the tumor microenvironment through the monocarboxylic acid transporter (MCT) on the cell membrane, and finally form a low glucose, high lactic acid microenvironment, such an environment is suitable for the survival of immunosuppressive cells. It can damage immune cells and help tumor cells escape immune surveillance (**Figure 2**) (24). Given these effects of lactic acid, they measure the level of lactic acid in tumor and throughout the body has shown promise as a marker for detecting and prognosis of certain cancers (25). Among patients with gastric cancer, the level of norepinephrine was significantly increased, while the norepinephrine degrading enzymes MAOA and MAOB were reduced considerably. Elevated norepinephrine is related to activated glycolysis, so the levels of MAOA and MAOB in tumor tissues are closely related to the prognosis of patients with PD-1 and other immunotherapy (26).However, these conclusions were based on the finding that tumor cells consume high levels of glucose. It is true that FDG-PET does not always provide the results clinicians expect. So, in 2021, a study upended 100 years of basic understanding of the metabolic model of cancer. The study used two PET tracers, one for glucose and one for glutamine, to separate the cancer tissue, using cell-surface marker proteins and flow cytometry to separate the tumor into cells and measure radioactivity in the cells. The results showed that in six cancers, including rectal, kidney and breast, myeloid immune cells absorbed the most glucose, followed by T cells and cancer cells. Cancer cells take up the most glutamine. Further studies revealed that the uptake of different nutrients by different cells depended on specific signaling pathways. So this study overturns 100 years of thinking that tumors suppress other immune cells by competing to get too much glucose. Instead, cells are metabolically reprogrammed to take in certain nutrients in response to specific cellular signaling pathways. This will help us further develop specific targeted therapies, as well as more effective PET methods that allow clinicians to better see where the tumor is (27).

It is worth noting that an effect called reverse Warburg seems to be different from traditional views. This study believes that fibroblasts in the tumor microenvironment exhibit high glycolytic activity. The lactic acid produced is released into the microenvironment through MCT1 and is taken up by tumor cells for oxidative stress phosphorylation(**Figure 2**) (28). The detection of MCT1 content in tumor stroma has also become a predictor of adverse outcomes in triple-negative breast cancer (29). However, such effects may only be manifested in certain types of cancers. In addition, tolerant DC cells have also been shown to weaken the role of T cells by releasing lactic acid and acting on T cells (30). Regardless of the Warburg or reverse Warburg effect, the high lactic acid in the tumor microenvironment is specific. It has reason to believe that this is the joint effect of multiple cells in the microenvironment.

Secondly, HIF-1 can promote epithelial-mesenchymal transition among cancers. Epithelial-Mesenchymal Transition (EMT) refers to the biological process of epithelial cells transforming into cells with a mesenchymal phenotype through specific procedures. The characteristics include the decrease of cell adhesion molecules, the up-regulation of vimentin and so on. The activation of HIF-1 can overexpress VASP, and VASP changes the EMT phenotype by activating AKT and ERK signals and promotes the metastasis of liver cancer *in vivo* and *in vitro (* 31). The latest research shows that HIF-1 mediates cervical cancer metastasis by directly targeting hCINAP (32). HIF-1 can also regulate long non-coding RNA, such as lncRNA RP11-390F4.3, and promote epithelial-mesenchymal transition (33).

Finally, HIF-1 is also involved in angiogenesis in tumor (34). In addition to VEGF, the mechanism of HIF-1 inducing angiogenesis can also be mediated by the SNHG1-miR-199a-3p-TFAM axis (35). In some other diseases, more and more evidence shows that microRNA also plays a vital role in the angiogenesis promoted by HIF-1 (36, 37).

In addition to activating HIF-1α signal, hypoxia also has other different effects. For example, hypoxia induces the expression of hyaluronic acid synthase and reduces the synthesis of hyaluronic acid degrading enzymes, resulting in high hyaluronic acid concentrations in hypoxia environments (38). Based on this phenomenon, a recent study used nanosystems to remotely enhance hyaluronidase activity, promote hyaluronic acid degradation, and reduce HIF-1α expression, alleviating the immunosuppressive tumor microenvironment (39).

Targeting hypoxic environments directly also seems like a good option, A study of microalgae to improve the hypoxic microenvironment and increase the sensitivity of tumors to radiation therapy demonstrates the broad range of tumor therapies (40).

## 3 Lipid metabolism in tumor microenvironment

The rapid growth of tumor cells requires a lot of energy, and glycolysis alone is certainly not enough. Therefore, lipid metabolism is essential for tumor cells, and lipids are not only the substrates for forming cell membranes; It can also undergo β oxidation and produce large amounts of ATP; It also acts as some of the second messengers involved the signalling axis of tumor development.

**Figure 3:**
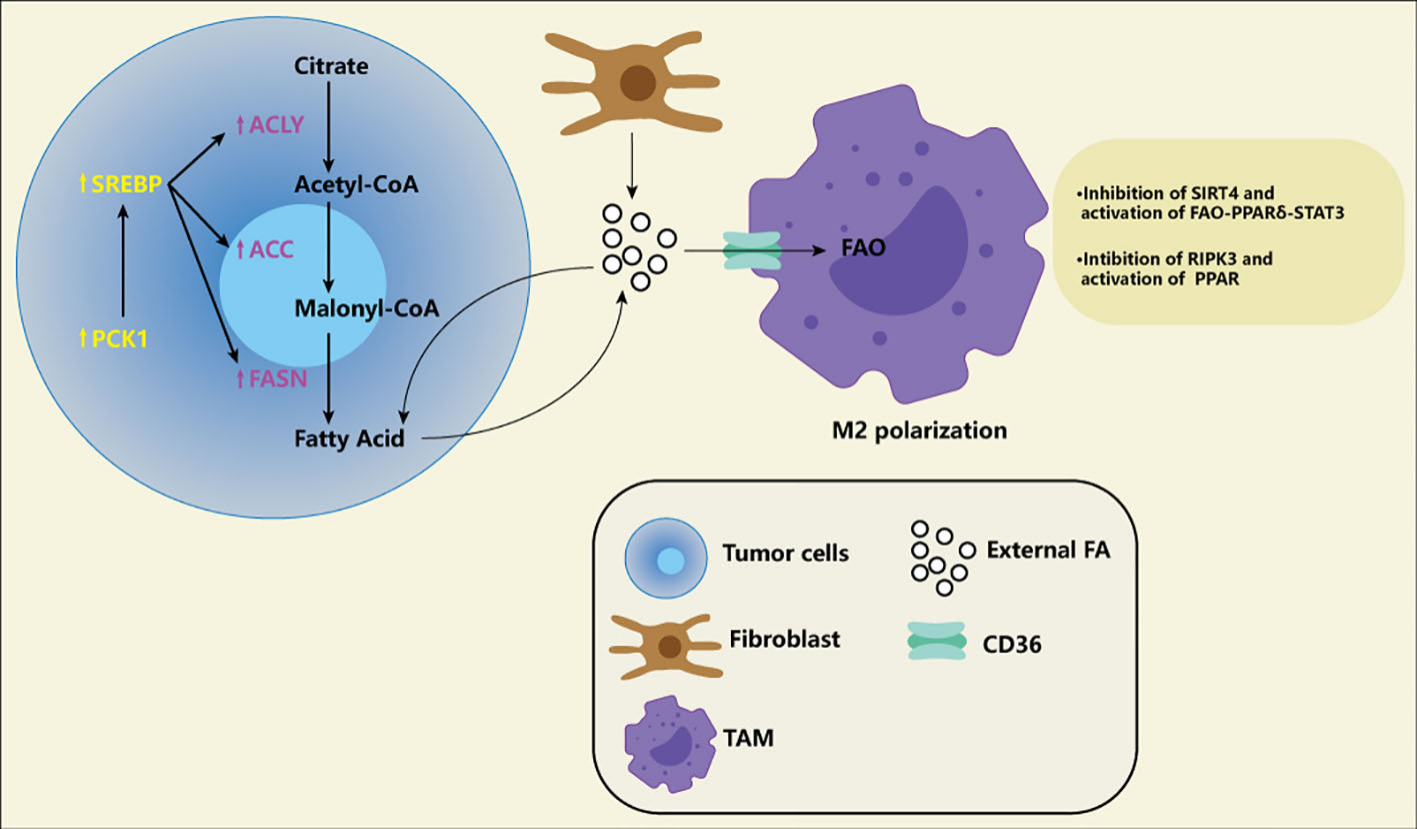
Fatty acid (FA) in the tumor microenvironment. The increase of FA exists in various tumor microenvironments. Tumor fibroblasts release a large of FA into the microenvironment, and some of the FA are absorbed by tumor cells for energy generation. Of course, tumor cells themselves also promote FA synthesis by increasing the activity of ACLY, ACC and FASN. Meanwhile, PCK1 induces FA synthesis through SREBP pathway, which is considered to be a specific way of tumor cells. FA produced by tumor cells can also be transported into the microenvironment and become part of the composition of exogenous FA. These exogenous FA are transported by CD36 receptors on the macrophages to activate FAO and promote M2-type polarization of macrophages. Macrophages themselves also adapt to this environment by down-regulation SIRT4 and RIPK3 and activating PPAR.

Studies have shown a high throughput of lipid metabolism in tumor cells, either through the uptake of lipids from the microenvironment or by improving their ability to synthesize lipids. Where do the rich lipids in the microenvironment come from? Fibroblasts in tumor tissue can synthesize more lipids and release them into the microenvironment (41). High protein expression associated with fatty acid intake, CD36, is associated with poor prognosis in patients with breast, ovarian, gastric, and prostate cancers (42). Key enzymes related to fatty acid synthesis and cholesterol synthesis, for example, long-chain acyl-CoA synthetase (ASCL), 1-aminocyclopropane-1-carboxylic acid (ACC), Fatty acid synthase (FASN), stearoyl-CoA desaturase (SCD), 3-hydroxy-3-methyl glutaryl coenzyme A reductase (HMGCR), Serine Proteinase (SM), several studies have shown the high expression of critical enzymes in cancer cells and promote the development of tumor (43–51). A new study has revealed the specific lipid anabolic mechanism of tumor cells, suggesting that phosphoenolpyruvate carboxylated kinase 1 (PCK1) can be phosphorylated to achieve protein kinase activity (**Figure 3**). PCK1 with protein kinase activity can phosphorylate INSIG1/2, which leads to impaired binding of INSIG1/2 with intracellular lipids, thus promoting the activation of the SREBP signaling pathway and lipid synthesis of tumor cells (52). This suggests that tumor cells may have a different lipid synthesis pathway from normal cells and also provides a new direction for us to target lipid metabolism. Finally, fatty acid oxidation also shows high activity in many cancers (53). Studies have shown that fatty acid oxidation can improve radioresistance in nasopharyngeal cancer and breast cancer (54, 55). A recent study suggests that fatty acid oxidation can not only improve tumor radioresistance but also mediate immune evasion of tumor cells through CD47 (56). In addition, it also knows that the metabolism of M2 macrophages and Tregs is more prone to fatty acid oxidation and oxidative phosphorylation. A recent single-cell-based study systematically delineated the heterogeneous population and different differentiation pathways of Tregs, the study found that Tregs had nine functional subgroups, FOXP3^hi^ subgroup with the strongest immunosuppressive function had high expression of fatty acid oxidation related genes (57).

The highly active lipid metabolism in the microenvironment is not only manifested in tumor cells. Immunosuppressive cells Such as Tregs and M2 macrophages are also believed to be involved in this process and thus consolidate their immunosuppressive ability.

## 4 Macrophages in the tumor microenvironment

Macrophages are the body’s “cleaners,” regulating the immune response to pathogens and maintaining tissue homeostasis. They can be polarized in a particular environment to perform a specific function. Polarized macrophages can be roughly divided into two categories: M1 type macrophages and M2 type macrophages. IFN-γ, LPS activation, produces M1-type macrophages; IL-4 and IL-13 evoke M2 macrophages (58). M1-type macrophages are pro-inflammatory cells, clearing pathogens by producing TNF-α, IL-6, IL-12, etc. In contrast, M2 macrophages inhibit inflammation by producing TGF-β, IL-10, promoting angiogenesis, tissue remodeling, and repair (59). Different subtypes of macrophages have other metabolic preferences. M1-type macrophages tend to use glycolysis rather than oxidative phosphorylation (60, 61). M2 macrophages showed increased oxidative phosphorylation and fatty acid oxidation flux (62, 63). M. de-Brito et al. showed that M2-like macrophages exhibit similar metabolic needs to M1-like macrophages, namely enhanced glycolysis and lactic acid production (64). However, Wang et al. showed that M2 macrophage differentiation does not depend on glycolysis as long as oxidative phosphorylation is active (65). Therefore, the different metabolic preferences of macrophages are still worthy of further study.

Tumor-associated macrophages (TAM) are macrophages involved in the formation of the tumor microenvironment. Tumor-associated macrophages are widely present in various tumor (66). TAM also shows a similar activation phenotype to macrophages and promotes tumor growth, invasion, metastasis, and drug resistance (67). So what exactly causes tumor-associated macrophages to be “bought” to have a role in tumor growth? More studies have shown that metabolites in the tumor microenvironment (TMA) play an essential role.

### 4.1 The effect of lactic acid on macrophages

Ohashi et al. showed that in squamous cell carcinoma of the head and neck, high lactic acid accumulation in the tumor microenvironment could promote The M2-type polarization of TAM (68), consistent with the different metabolic preferences of varying macrophage subtypes mentioned before, but the specific mechanism is not precise. In breast cancer, Mu et al. showed that lactic acid promoted M2-type polarization of macrophages through the ERK-STAT3 pathway (69).

Chen et al. showed that a high lactic acid concentration in the tumor microenvironment induces M2-type polarization by activating macrophage Gpr132 (70). In addition, a recent study showed that the lactyl group extracted from lactic acid can be used for post-translational modification of histones, resulting in increased expression of M2 marker genes, such as IL6 and arg1 (71).The M2-type polarization of macrophages induced by lactic acid may occur through other molecular mechanisms in different tumor. Therefore, it is crucial to understand the influence mechanism of lactic acid on macrophage typing in various tumor.

As previously mentioned, M2-type macrophages are mainly involved in immunosuppression and angiogenesis promotion. More and more studies have shown that M2-type macrophages can help tumor cells get rid of the “monitoring” of the immune system through various ways.

Shan et al. found that lactic acid-induced high expression of PD-L1 in M2-type macrophages promotes the immune escape of tumor cells, and HIF-1α knockdown in macrophages significantly reduces the expression of PD-L1, suggesting that HIF-1α plays a vital role in the immunosuppression of macrophages (72).

Zhang et al. showed that activated M2-type macrophages promote breast cancer metastasis through the CCL17/CCR4/mTORC1 axis (73). In liver cancer and pancreatic cancer, lactic acid-induced reactive oxygen species can activate Nrf2 of macrophages and promote VEGF expression, which is conducive to angiogenesis during tumor growth and improves epithelial-mesenchymal transformation of tumor (74). In lung cancer and melanoma, lactic acid has also been found to activate mTORC1 and inhibit Feb-mediated lysosomal membrane protein (ATP6V0d2) expression, thereby alleviating HIF-2α degradation and leading to continuous HIF-2α -mediated VEGF production (75). These studies provide new targets for the inhibition of angiogenesis in tumor. Of course, the high content of VEGF can promote the generation of blood vessels and the accumulation of Tregs in the tumor microenvironment (76), making the “accomplice” of tumor cells in the microenvironment more powerful. Meanwhile, TNF-α secreted by TAM can promote glycolysis of tumor cells, and the increased AMPK and peroxisome proliferator-activated receptor gamma coactivator1-α in TAM can promote hypoxia of tumor cells (77). This creates a vicious cycle, making it easier for tumor cells to evade the immune system.

Although macrophages become accomplices in tumor immune escape due to the “temptation” of lactic acid secretion by tumor, it can still regulate macrophages by inhibiting lactic acid production and influencing the pathway of lactic acid action on macrophages.

Chung et al. found that MachilinA functions as a competitive inhibitor by blocking the NADH binding site of LDHA, reducing the proportion of M2-type macrophages and angiogenesis in colorectal cancer, breast cancer, lung cancer, and liver cancer (78). In gastric cancer, either CHC, an inhibitor of MCT1, or HIF-1α knockout can significantly reduce the M2 phenotype of macrophages (79).

### 4.2 Effects of lipid substances on macrophages

#### 4.2.1 The fatty acids

The need for fatty acid metabolism in different subtypes of macrophages is controversial, especially in M2-type macrophages. It has been suggested that M2-type macrophages are more prone to fatty acid oxidation and oxidative phosphorylation; It has also been suggested that M2-type macrophages prefer glycolysis as their means of production. However, a growing number of experiments seem to support the first view. Liu et al. suggested that S100A4 can promote the M2-type polarization of macrophages. The molecular mechanism is that S100A4 can make macrophages highly express CD36 (a fatty acid transporter located on the cell membrane) by activating PPAR-γ and promoting heavy acid absorption and fatty acid oxidation of macrophages (80).Similarly, Li et al. found that SIRT4 is generally low expressed in tumor-associated macrophages of liver cancer, and down-regulation of SIRT4 can enhance fatty acid oxidation of macrophages through the FAO-PPARδ-STAT3 axis, resulting in more transformation of macrophages into The M2 subtype. Notably, the silencing of SIRT4 in macrophages can promote the proliferation, migration, and invasion of HCC cells by increasing the secretion of IL-6. It can boost the apoptosis of M1-type macrophages by enhancing the production of IL-10, which can better deteriorate the tumor immune microenvironment and promote the occurrence and development of liver cancer (81). Wu et al. also pointed out the low expression of RIPK3 by TAM in liver cancer. The loss of RIPK3 can also promote fatty acid oxidation and M2-type polarization of macrophages by activating PPAR(**Figure 3**). RIPK3 can inhibit PPAR through ROS-caspasl signalling (82). These findings all seem to indicate that increased peroxisome proliferator-activated receptor-dependent fatty acid oxidation can induce M2 polarization in macrophages, and there may be many upstream factors that play different roles in different cancer. Therefore, the study of PPAR and its pathway seems to provide us with a new target to improve the tumor microenvironment by changing the classification of tumor-associated macrophages.

Many other studies have also demonstrated that fatty acids are essential for the polarization of tumor-associated macrophages. Similar to the findings of Liu et al., Su et al. also found that tumor-associated macrophages can accumulate super lipids through CD36 in lung cancer and breast cancer and promote fatty acid oxidation and tumorigenesis (83). Wu et al. found that lipid droplet-derived fatty acids, predominantly unsaturated oleic acid, in macrophages can promote the differentiation of M2 subtype in colon cancer and fibrosarcoma, in which mTORC2 is an important molecule (84). It has also been proved that fatty acid oxidation plays an essential role in M2-type macrophages and promotes the migration of liver cancer by enhancing the secretion of IL-1β (85). Therefore, more evidence indicates that M2 macrophages are more dependent on fatty acid oxidation, and glycolysis is just a replacement mode of M2-type macrophages when the mitochondrial oxidative respiratory chain is damaged (65).

Interestingly, macrophages can improve the intake of exogenous fatty acids through fatty acid transporters such as CD36 and make themselves highly accumulate fatty acids by enhancing their fatty acid synthesis. Limet al. suggested that Tregs could indirectly maintain metabolic adaptation and mitochondrial integrity of M2-like macrophages by inhibiting IFN-γ secretion of CD8+T cells and promoting SREBP1 mediated fatty acid synthesis in M2-like macrophages (86). Similarly, TAM in TC and NB can also promote tumor development by increasing fatty acid synthesis (87). Notably, in this study, macrophages promoted their inflammatory phenotypes by enhancing their fatty acid synthesis, contrary to the previous report that macrophages differentiated into the anti-inflammatory M2 phenotype by accumulating fatty acids and promoting fatty acid oxidation. That may be because inflammation plays an utterly opposite role in different tumor.In some cases, inflammation promotes tumor development; And in some cases, inflammation inhibits tumor growth. Therefore, it is worth our attention whether the accumulation of fatty acids promotes the occurrence and development of tumor in different tumor tissues. This seems to be a problem for those trying to improve the efficacy of immunotherapy by enhancing fatty acid metabolism.

Fatty acid metabolism is significant for the classification of tumor-associated macrophages, so can be targeted fatty acid therapy play an influential role? Wu et al. found that DGAT plays a vital role in the process of rich acid transfer to intracellular lipid droplets, and its inhibitors can well inhibit the growth of colon cancer in mice (84). In patients with gastric cancer, lipid accumulation of TAM can promote M2-type polarization of macrophages through PI3K-γ, and IPI549, as a selective inhibitor of PI3K-γ, can reverse this process (88). A high dose of dexamethasone also seems to have such effects. Studies have found that a high amount of dexamethasone can significantly inhibit the expression of exogenous fatty acid ingestion related proteins and heavy acid oxidation related genes and convert M2-type macrophages into M1-type macrophages, successfully improving the tumor microenvironment (89). Notably, Su et al. found that myeloma developed in the myeloma mouse model and led to death in the control mice. The CD36 KO mice survived (83). More importantly, through the collation of clinical data, this study showed that CD36 was highly expressed on the surface of tumor-associated macrophages in lung cancer and breast cancer patients and was significantly higher than other normal tissue macrophages. This suggests that CD36 can be used as a specific target for fatty acid metabolism of macrophages in lung cancer and breast cancer to improve the tumor microenvironment while reducing the influence of M2-type macrophages in other normal tissues, thus reducing the damage to the autoimmune system. Therefore, this suggests that when studying the relationship between fatty acids and macrophage typing, it should pay attention to the specific molecular mechanism and evaluate whether the expression of these molecules is specific to macrophages in other normal tissues to minimize the impact on autoimmunity.

#### 4.2.2 Cholesterol

In addition to fatty acids, cholesterol with the cyclopentane poly-hydro phenanthrene structure appears to play an essential role in the M2-type polarization of macrophages. Goossens et al. found that in ovarian cancer, tumor cells can drive the cholesterol effection of TAM through the release of hyaluronic acid, which promotes IL-4-induced macrophage reprogramming (towards M2-type differentiation). The specific molecular mechanism requires STAT6 phosphorylation and PI3K-MTORC2-Akt activation (90). Similar to Su et al. ‘s study, phosphorylation of STAT6 seems to play an essential role in the M2 typing of macrophages. Many studies have proved that inhibition of STAT6 can reverse the M2-type polarization of macrophages and inhibit the occurrence and development of tumor (91–93). In addition to fatty acids, cholesterol with the cyclopentane poly-hydro phenanthrene structure appears to play an essential role in the M2-type polarization of macrophages (94).

#### 4.2.3 Prostaglandins

In glioblastoma, tumor cells can activate MGLL through ARS2, and monoacylglycerol lipase (MAGL) is the translation product of MGLL, which can produce PGE2 to promote M2 polarization (95). Interestingly, MGLL deficiency in macrophages promotes CB2/TLR-4 dependent macrophage polarization in mouse colon cancer (96).

## 5 Tregs cells in the tumor microenvironment

Tregs cells are the essential immunosuppressive cells in the tumor microenvironment because Tregs can inhibit various immune cells, such as T cells, DC cells, etc. Tregs in tumor tissues is a group of cells expressing Foxp3, CD4, and high expression of CD25. Interestingly, CD25, as the α chain of the IL-2 receptor, can effectively bind IL-2. At the same time, Tregs cells themselves expressed less IL-2. More importantly, IL-2 is an essential substance for effector T cells to produce immune function. Therefore, such a high expression of CD25 in Tregs cells can effectively reduce the IL-2 utilized by effector T cells in tumor tissues and reduce the immune activity of effector T cells (76). Of course, Tregs can also achieve immunosuppressive function in a variety of other ways. For example, Tregs can kill effector T cells directly by the FASL-FAS pathway or by producing grease B and perforin (97, 98). The proportion of Tregs to effector T cells in the tumor microenvironment also affects prognosis and clinical treatment (99–101). In general, Tregs infiltration is negatively correlated with prognosis. However, in some tumor, Tregs density was positively associated with prognosis (102, 103). This may be because inflammation plays a different role in different tumor.

Many therapeutic approaches aimed at reducing Tregs in the tumor microenvironment have not achieved good results. On the one hand, targeting Tregs is likely to damage one’s immune system because Tregs plays an essential role in regulating immune balance in normal tissues. On the other hand, there seems to be a “mysterious force” in the tumor microenvironment that deliberately amplifies Tregs strength. Therefore, finding specific targets targeting Tregs in the tumor microenvironment or revealing this mysterious force is essential for current research.

A growing body of research suggests that tumor’ unique metabolisms and metabolites seem to be part of this mysterious force.

### 5.1 Effects of lactic acid on Tregs

How low glucose and high lactate environments in tumor are created is not explained here. The activation and function of T cells also depend on glycolysis for energy (104). It’s not hard to imagine that T cells and tumor cells would have to compete with glucose in a low-glucose environment to complete glycolysis while simultaneously removing lactic acid, an intermediate product of glycolysis, from the body. On the one hand, T cells are much smaller than tumor cells, so can they successfully compete for glucose? On the other hand, in the tumor microenvironment with a high concentration of lactic acid, MCT-1 on the surface of T cells is inhibited, and T cells cannot expel lactic acid generated in the body, resulting in inhibition of metabolism, proliferation, and functional decline (105).

However, Tregs cells did not seem to be affected by low glucose, high lactate environment. This may be related to its metabolic reprogramming. It has been reported that Foxp3 can help Tregs adapt to this environment. Foxp3 can not only help Tregs turn to rely on oxidative phosphorylation for energy supply; It also inhibits Myc (a transcription factor that plays an essential role in upregulating glycolysis in activated T cells) and glycolysis (by restricting the direction of lactate dehydrogenase, which prevents pyruvate from producing lactate through LDH) (106). In conclusion, in such an environment of low glucose and high lactic acid, the metabolism of effector T cells is inhibited. At the same time, Tregs shows metabolic adaptability and increased activity by mitochondrial oxidative phosphorylation (107).

Therefore, can glycolysis targeted at tumor cells or lactic acid uptake targeted at Tregs achieve good results in immunotherapy? Recently, a study showed that inhibition of tumor glycolysis contributes to the therapeutic effect of CTLA-4 blockade, and the combination of CTLA-4 blockade and tumor glycolysis inhibitors may be considered (108). Another study suggested that elimination of ALKBH5 (RNA m6R demethylase) reduced the expression of MCT4/SLC16a3 on melanoma cells, reduced the accumulation of lactic acid in TME, and weakened the immunosuppressive function of Tregs (109). At the same time, this study also proved that ALKBH5 inhibition could improve the effect of anti-PD-L1. Similar to this study, Liu et al. found that FTO protein, also RNA m6R demethylase, allows glycolysis of tumor cells by upregulating transcription factors C-Jun, JunB, and C/EBP β. Inhibition of FTO can significantly reduce lactic acid produced by the glycolysis of tumor cells. The research team also developed an effective FTO molecular inhibitor, Dac51. It provides a new therapeutic target for tumor glycolysis (110).

However, both blocking tumor cell glycolysis and reducing Tregs lactic acid uptake raises the question of whether the use of inhibitors, many of which lack specificity, can cause autoimmune damage. Happily, Greg M. Delgoffe’s team found that inhibition of the expression of MCT1 monocarboxylic acid transporter on Tregs could significantly reduce the intake of lactic acid in Tregs while weakening the inhibitory ability of Tregs. Because they co-operated with anti-PD-1 therapy by triggering MCT1 deletion in Tregs cells, this procedure resulted in complete tumor regression in 37.5% of B16-bearing mice. More importantly, they found that the loss of MCT1 appears to be optional for Tregs cell survival and function but may impact tumor tissue rich in lactic acid (111). In addition to specificity in inhibitory targets, it can also choose to innovate in pharmaceutical materials. Recently, a new nanomaterial that encapsulates the MCT1 inhibitor AZD3965 has been reported, which can be released to target MCT1 on the surface of tumor cells when the ph of the external environment is lowered to reduce lactic acid accumulation in TME, improve the microenvironment and improve the efficacy of immunotherapy (112). The benefit of this material is that when used with less specific inhibitors, it can target the tumor microenvironment with high lactate and thus work, reducing the risk of autoimmune damage.

### 5.2 Effects of lipid substances on Tregs

#### 5.2.1 The fatty acids

Like M2-type macrophages, Tregs cells also rely on mitochondrial oxidative phosphorylation, and fatty acid oxidation seems to provide sufficient “material” for oxidative phosphorylation of Tregs cells. Such metabolic change of Tregs cells also appears to be affected by HIF-1α (113). Similarly, Tregs has two ways to obtain fatty acids: from tumor microenvironment; It depends on its fatty acid synthesis.

In gastric cancer, the Y42 mutation of tumor RHOA was observed, which increased the production of free fatty acids in GC cells by activating the PI3K-AkT-mTOR signalling pathway. The resulting fatty acids provide more energy for Tregs. Combined use of PI3K inhibitors and PD-1 inhibitors was found to significantly reduce resistance to PD-1 in RHOA Y42 mutated gastric cancer. This provides a new therapeutic idea for patients who are resistant to immune checkpoint inhibition (114). In another study, researchers compared Tregs in breast cancer with Tregs in PBMC. They found that Tregs in breast cancer expressed high levels of the fatty acid transporter CD36, which has also been demonstrated in other cancers. This also seems to indicate that Tregs consolidate their immunosuppressive function by ingesting exogenous fatty acids. Because the researchers combined the CD36 knockout mice with a PD-1 inhibitor, they found that the survival of the mice in this group was significantly improved. In addition, CD36 was found to activate PARP-β signaling and enhance mitochondrial function, further helping Tregs adapt to TME. Notably, CD36 defects did not damage the immune system and did not affect Foxp3 expression in Tregs cells (115). This provides a new target for improving the tumor immune microenvironment. It can consider whether CD36 is highly expressed in Tregs and M2 macrophages in the same tumor environment and whether this can play a dual role.

Interestingly, however, when inhibited FABP5 (a fatty acid-binding protein) on Tregs, it found that Tregs mitochondria were damaged, and oxidative phosphorylation decreased. Still, Tregs released mtDNA and induced increased IL-10 expression through cGAS-STING dependent I-IFN signaling. It promotes Tregs inhibition. This seems to contradict the previous discussion (116). However, the experiment did not examine whether long-lived Tregs were in stock or whether IL-10 release decreased over time.

On the other hand, Tregs in the tumor microenvironment are self-reliant and seem to be trying to generate enough energy for themselves through their fatty acid synthesis. Lim et al. found that SREBP1/2 protein expression in regulatory T cells was significantly increased in melanoma, breast cancer, and head and neck squamous cell carcinoma microenvironments than in other parts of the body. When they knocked out the SCAP gene, an upstream protein of SREBP, in mouse models, the mice became more resistant to transplanted tumor and could even fully recognize colorectal cancer cells. Of course, the combination of PD-1 inhibitors has also shown surprising results in melanoma. Further studies found that SCAP knockout could affect the production of fatty acid synthase FASN, and Tregs without FASN could not be activated, thus losing their immunosuppressive function. More happily, they found that SCAP knockout mice did not develop autoimmune diseases, and the PROPORTION of CD4+/CD8+ T cells in other tissues remained regular, which seems to indicate that SCAP knockout does not affect T cells in other normal tissues, which is a specific target (86).

#### 5.2.2 Cholesterol

The main metabolic pathway of cholesterol is the MVA pathway. Tregs cells can up-regulate the MVA pathway through LKB1, thus maintaining their own function and stability (117). The MVA pathway in Tregs seems to be inhibited in the tumor microenvironment, however, this does not affect the immunosuppressive function of Tregs itself. It may be related to tumor cells themselves through high expression of MVA pathway, the resulting FPP synthesizes large amounts of Kynurenine through RAS-ERK-STAT3-IDO and promotes the expansion of Tregs (118).

Cholesterol may have more objections to the function of CD8+ T cells than Tregs. One study found that an increase in free cholesterol in CD8+ T cells resulted in more mature and intact synapses on cells (119). Another recent study showed that higher cholesterol levels were associated with higher levels of PD-1,LAG-3,TIM-3,2B4 and other immune checkpoints (120). This may also depend on the heterogeneity of the tumor. Therefore, further research is needed on the role of cholesterol metabolism in tumor microenvironment.

## 6 NK cells in the tumor microenvironment

NK cells are a group of large particles of lymphocytes, which contain granzymes and perforin, responsible for NK cells killing (121). NK cells are also an integral part of the immune system, responsible for clearing pathogens and tumor. A growing number of studies have also shown that damaged NK cells or lack of NK cells increase the risk of cancer in both patients and mouse models (122). This seems to suggest that NK cells also play an essential role in the process of tumor resistance.

Therefore, we must first understand how NK cells are damaged in the tumor environment to provide a solid foundation for immunotherapy based on NK cells. Studies have shown that tumor cells can release TGF-β (123), PGE2 (124), IDO, adenosine acid (125, 126), and IL-10 (127, 128) to damage the function of NK cells and avoid attacks from NK cells. These appear to be just some of how tumor cells consume NK, and some recent studies have shown that lactic acid released by tumor cells can also severely damage NK cells.

Husain et al. suggested that lactic acid could inhibit the killing effect of NK cells *in vitro*. Secondly, injection of LDH-A-deficient pancreatic cancer cells into mouse models significantly enhanced the cytotoxic activity of NK cells (129). However, some studies suggest that the cytotoxic activity of NK cells is inhibited *in vivo*, which is not caused by lactic acidosis, but the specific mechanism is still unclear (130). Another study also showed that tumor with low LDHA expression in melanoma grew more slowly, with increased NK cells infiltration and increased IFN-γ release (131). Indeed, it facilitated the decrease in IFN-γ release from NK cells due to acidosis when adopted a systemically buffered alkalization approach (130).

Lactic acid produced by tumor cells results in the loss of function of tumor-infiltrating NK cells and damages the original “resident” NK cells in metastatic tissues, providing a perfect environment for the arrival of tumor. Harmon et al. found that liver metastasis of colorectal cancer can lead to depletion of NK cells in the liver itself, which is due to the production of large amounts of lactic acid by tumor cells, the reduction of pH value in the environment, and the NK cells apoptosis caused by mitochondrial damage of NK cells through ROS (132).

## 7 Immunotherapy and targeting the tumor microenvironment

Immunotherapy has become a powerful weapon against cancer. The success of ipilimumab, which targets PD-1/PD-L1 and CTLA-4, has also brought hope to many cancer patients. However, not all patients are sensitive to immunotherapy, which leads to the limitations of immunotherapy in the application process. Then what causes such difference in immunotherapy has become the focus and difficulty of research. More and more studies have shown that the tumor microenvironment affects the effect of immunotherapy, including cytokines and metabolites in the microenvironment. In triple-negative breast cancer, different subtypes were found to have different sensitivity to other metabolic inhibitors. MPS1 was more sensitive to inhibiting fatty acid synthesis, while MPS2 was more sensitive to inhibiting glycolysis. This suggests that various tumor may have metabolic preferences that need to be adapted to local conditions. However, it is encouraging to note that targeting metabolism in the tumor microenvironment appears adjunct to immunotherapy (133).

### 7.1 Targeted HIF - 1

In the hypoxia environment, the high expression of HIF-1 in glioma cells affects the increased expression of PD-L1, and the combination of HIF-1 inhibitor and PD-L1 inhibitor can significantly improve the efficacy (134). Before this, HIF-1 inhibitors have been used as a method of tumor therapy due to the effect of HIF-1 on tumor development. However, recent studies have found that HIF-1 signaling seems to be related to the high expression of PD-L1 mediated immunotherapy tolerance on tumor cells. In non-small cell lung cancer, EZH2 upregulates the expression of PD-L1 through HIF-1α, so the combination of HIF-1 inhibitors and immunotherapy may have a more substantial therapeutic effect (135). As an inhibitor of HIF-1α and HIF-1β dimer, acriflavine can significantly improve the efficacy of anti-PD-L1 immunotherapy (136). The HIF inhibitor PT2385, completed phase 1 clinical trials in clear cell renal cell carcinoma, also showed better efficacy when combined with a PD-L1 inhibitor (137).

Ginseng diol, a Chinese herbal component, also showed inhibition of HIF-1α formation and blocked the interaction between HIF-1α and STAT3, inhibiting the expression of PD-L1 in colon cancer cells, and also showed minor cytotoxicity in cell experiments (138). Another Chinese herb, turmeric, and turmeric has been found to have a similar effect on liver cancer (139). However, it is unclear whether these herbs could have a more powerful influence in conjunction with immunotherapy.

### 7.2 Target glucose metabolism

In some patients, resistance to immune checkpoint inhibition is primarily determined by lactic acid levels in the tumor microenvironment. Lactic acid can help immunosuppressive cells survive and perform their functions in the microenvironment, and improve the expression of PD-L1 on tumor cells. A recent study found that upregulation of PD-L1 in nasopharyngeal carcinoma cells is associated with active glycolysis, and sirilimarine (A drug used in hepatitis, cirrhosis, and liver protection) interferes with HIF-1α/LDH-A mediated glucose metabolism and shifts to mitochondrial oxidative phosphorylation, reducing PD-L1 expression (140).

Metformin, a clinical drug used in patients with type 2 diabetes, affects glycolysis in various cancers (myeloma, liver cancer) by interfering with HIF-1 signaling (141, 142). Meanwhile, metformin has also been reported to significantly improve the immune microenvironment in type 2 diabetes and colorectal cancer patients (143). Metformin in combination with immune checkpoint inhibitors has also been shown to be more effective in melanoma (144). Conversely, metformin is also thought to inhibit tumor cell response to paclitaxel by inducing lactic acidosis (141). Therefore, whether metformin can improve tumor microenvironment and enhance immunotherapy efficacy through targeted metabolism still needs a systematic study.

### 7.3 Target lipid metabolism

The activity of lipid metabolism in tumor microenvironment mentioned above is increased, including the fatty acid synthesis, fatty acid oxidation and the MVA pathway of cholesterol synthesis.

FASN is a key enzyme in fatty acid synthesis. Studies have shown that FASN inhibition both limits tumor-cell migration and invasiveness, and increases tumor sensitivity to drug therapy (145, 146). Recent studies have found that FASN can also be used as an indicator of anti-CTLA-4 and anti-PD-1 therapy in BC patients (147).

A fatty acid oxidation inhibitor, namely ranolazine which showed reduced colony-forming activity of GBM combined with DCA (148).

Lipid-lowering statins is an inhibitor of cholesterol synthesis which appear to improve immunotherapy in non-small cell lung cancer, but further studies are needed to confirm this (149). Because there are different opinions about the effects of statins. Several studies have shown that statins improve first-line chemotherapy survival in metastatic pancreatic cancer and multiple myeloma patients (150, 151). In contrast, the addition of statins did not benefit patients with advanced liver cancer (152). Therefore, lipid metabolism has a long way to go than targeting glucose metabolism.

PCSK9, a protein that plays a crucial role in cholesterol regulation. One study showed that inhibiting this protein allowed tumor cells to express more MHC-1, increasing T cell infiltration and boosting immune checkpoint inhibition. However, this relied on a method independent of cholesterol metabolism (153).

In addition, PPARα can inhibit DC dysfunction induced by lipid-rich exosomes secreted by tumor cells and improve the effect of immunotherapy (154).

## 8 Nanomaterials

As the role of the tumor microenvironment in tumor genesis and development has been continuously discovered, some drugs targeting glucose metabolism and lipid metabolism have also been found to improve the efficacy of immunotherapy. Therefore, nanomaterials are gradually added to the family of immunotherapy, which can more accurately target the tumor microenvironment and minimize systemic side effects caused by immunotherapy.

For glucose metabolism: Li et al. loaded chemotherapy drugs HCPT and siMCT-4 on PEG-CDM modified GSH responsive hollow mesoporous organic silica nanoplatforms for targeted tumor therapy, which can effectively induce apoptosis of tumor cells, reduce lactic acid concentration in the microenvironment, improve macrophage M2/M1, and restore the function of efficient T cells (155). In another study, they designed a ph-responsive coated nanoparticle that targets the tumor microenvironment through metformin and siFGL1 to collaborate with immunotherapy against breast cancer (156).

For lipid metabolism: Kim et al. activate lipid metabolic drug molecules packages amid amphiphilic poly glutamic acid nanoparticles and adopt CD3ef resistance (ab ‘) 2 pieces modified nanoparticles surface of T cells to achieve targeted transport, protection of T cells in the microenvironment of the lack of glucose metabolism difficulties, restore the function of T cells, enhance the effect of immunotherapy against PD - 1 (157).

## 9 Ferroptosis and tumor microenvironment

Ferroptosis, a new way of cell death first proposed in 2012 (158). Unlike autophagy and apoptosis, ferroptosis is iron-induced reactive oxygen-dependent cell death, mainly characterized by the disappearance of the mitochondrial crest and the rupture of mitochondria and mitochondrial outer membrane. Ferroptosis occurs through lipid peroxidation and oxidative stress in cells (159).

As the understanding of ferroptosis continues to grow, it increasingly believe that ferroptosis may be an essential way to inhibit cancer. In addition, some genes related to ferroptosis have been proved to be features for evaluating prognosis and the immune microenvironment in a variety of cancers (colon cancer, head, and neck squamous cell carcinoma, lung adenocarcinoma) (160–162). New studies have found that lncRNA related to ferroptosis can also be used as markers of prognosis and the immune microenvironment in breast cancer (163).

Is there a potential link between ferroptosis and tumor microenvironment? Cancer cells, Tregs, and M2 macrophages all show enhanced fatty acid oxidation in the tumor environment, but the incidence of ferroptosis is not high in these cells. So what is the reason to protect these cells from the damage of reactive oxygen species? The typical regulatory mechanism of ferroptosis is mediated by glutathione peroxidase GPX4 (164). Therefore, inhibition of GPX4 to induce ferroptosis in cancer cells may be a therapeutic strategy for some tumor, just as RSL3 inhibition of GPX4 induces ferroptosis in colon cancer (165). Of course, inhibition of GPX4 upstream, such as the cystine transporter SLC7A11, also seems to contribute to ferroptosis. Because cystine is taken up by cells *via* SLC7A11, it is reduced to cysteine in the cell and participates in glutathione formation. GPX4 is responsible for understanding the toxicity of reactive oxygen species in a glutathione-dependent manner. Studies have shown that SLC7A11 inhibition can also interfere with GPX4 protein translation by inhibiting the Rag-mTORC1-4EBPs signaling pathway, independent of glutathione (166). However, GPX4 inhibitors have different responses in different cancer cells (167), suggesting that other mechanisms regulate ferroptosis in different cells. Interestingly, it was found that high-density cells were more resistant to ferroptosis after GPX4 inhibition; The specific mechanism shows that this effect is generated through E-cadherin-mediated cell-cell contacts. Such intercellular interaction activates the Hippo pathway through NF2/Merlin tumor suppressor, thus inhibiting the transcriptional activity of YAP, and there are many genes regulating ferroptosis which is the target genes of YAP (168). A protein named FSP1 is considered to be a novel signal for inhibiting ferroptosis independent of the classical GPX4 pathway, and FSP1 inhibits ferroptosis by reducing lipid peroxidation in CoQ10 tissues. In lung cancer, the presence of FSP1 maintains the growth of lung cancer cells when GPX4 is inactivated, so an apparent targeting of FSP1 seems to be a new target for cancer therapy, the study said (169, 170). GPX4 exists in the cytoplasm, SLC7A11 exists in the cell membrane, and in mitochondria, dihydroorotate dehydrogenase DHODH resists ferroptosis by regulating dihydro ubiquinone production in mitochondria (171). In addition, SCD1 and FABP4 are also thought to inhibit ferroptosis in tumor cells by inducing desaturation of fatty acids which is essential for tumor relapse in response to tyrosine kinase inhibitors (TKI) and chemotherapy (172).

It is noteworthy that lactic acid in the tumor microenvironment can help HCC cells resist oxidative stress-induced lipid peroxidation through the HCAR1/MCT1-AMPK-SREBP1-SCD1 pathway (173). This gives us more reason to believe that lactic acid is not just a waste product of tumor cells’ metabolic adaptation. In addition, activation of the AMPK pathway can reduce the polyunsaturated fatty acid synthesis and inhibit ferroptosis by inhibiting ACC (174).

Tumor cells can inhibit ferroptosis through various mechanisms, while effector T cells, main glycolysis, are subjected to ferroptosis. Studies have shown that excessive cholesterol in the tumor microenvironment increases the expression of CD36 on effector T cells. In contrast, high expression of CD36 on T cells can make T cells absorb more fatty acids from the tumor microenvironment, induce lipid peroxidation and ferroptosis, and reduce the function of T cells and the production of toxic cytokines. CD36 blocking or ferroptosis inhibition on T cells can reshape the immune function of T cells and, more importantly, can be better combined with PD-L1 inhibitors (175, 176).

However, it is interesting that CD8+T cells can down-regulate cystine transporter expression on the tumor cell surface and promote ferroptosis by releasing IFN-γ (177). The specific mechanism was found in subsequent studies, because IFN-γ alone did not induce ferroptosis in tumor cells *in vitro*, so the researchers hypothesized that IFN-γ could act selectively with arachidonic acid. IFN-γ regulates the expression of ACSL4 (Acyl-CoA synthetase long chain family member 4) directly through STAT1-IRF1 signaling pathway, while arachidonic acid induces ferroptosis in tumor cells through preferential integration of ACSL4 into phospholipids containing C16 and C18 acyl chains. Finally, arachidonic acid supplementation inhibited tumor progression *in vivo* and synergized with the antitumor effects of PD-L1 inhibitors in immunocompetent mice (178). It provides a new basis for immunotherapy combined with targeting ferroptosis. In this study, in immunologically normal mice, the tumor size of the ASCL4 knockout model was significantly larger than that of the wild-type mice, and there were also fewer T cells in the tumor microenvironment, suggesting that ferroptosis of tumor cells can lead to the release and spread of tumor antigens, thereby promoting immunity. This finding is consistent with another finding that ferroptotic cells are immunogenic and contribute to tumor immune activation (179). All these conclusions seem to tell us that there is no harm in inducing ferroptosis of tumor cells.

However, studies have demonstrated that in pancreatic cancer, ferroptosis of tumor cells can release KARSG12D, which is packaged into exosomes and taken up by macrophages through the AGER pathway. KARSG12D promotes M2-type polarization of macrophages through STAT3-mediated fatty acid oxidation (180). In addition, an animal study also demonstrated that loss of GPX4 in mice with high iron diet and pancreatic cancer accelerated the development of pancreatic ductal adenocarcinoma mediated by Kras mutations through STING1/TMEM173 pathway (181). This suggests we by inducing tumor cell death treatment there is a lot of unknowns, based on the heterogeneity of tumor itself and the difference of various components in tumor microenvironment, it still needs to set up a complete database in order to analysis the differences between the various forms of cancer, and find out each tumor is the most suitable immune treatment measures.

## Conclusion

In summary, it still have a long way to go explain various phenomena in the tumor microenvironment and their influence on tumor development. It can’t be sure, for example, which fatty acids play a role in the microenvironment, or whether it depends on the characteristics of the tumor itself? Because of the heterogeneity of tumor, many studies may seem contradictory, but that is understandable. Of course, in addition to glucose metabolism and lipid metabolism, amino acid metabolism and frontal nucleotide metabolism also play an essential role in forming a microenvironment. Therefore, It should consider the effects of various metabolic factors on the tumor microenvironment in multiple aspects. It is worth paying attention to tumor heterogeneity, including the location of tumor organs and tissues. And whether metabolic tendencies change as the tumor progresses.

Finally, It should also develop effective and practical “weapons” that can predict the state of tumor microenvironment in clinical treatment so that clinicians can accurately and conveniently take appropriate adjuvant immunotherapy based on such prediction so that more cancer patients can improve their sensitivity to immunotherapy.

## Author contributions

ZZ wrote this manuscript. YH, YC, ZC, YZ, MC, and WX revised this manuscript. All authors contributed to the article and approved the submitted version.

## Funding

This work was supported by the Medical and Health Project Zhejiang Provincial Department of Health (2023KY1273) in the collection, analysis, and in the decision to submit the article for publication.

## Conflict of interest

The authors declare that the research was conducted in the absence of any commercial or financial relationships that could be construed as a potential conflict of interest.

## Publisher’s note

All claims expressed in this article are solely those of the authors and do not necessarily represent those of their affiliated organizations, or those of the publisher, the editors and the reviewers. Any product that may be evaluated in this article, or claim that may be made by its manufacturer, is not guaranteed or endorsed by the publisher.
